# Use of a New Ziprasidone-Selective Electrode in Mixed Solvents and Its Application in the Analysis of Pharmaceuticals and Biological Fluids

**DOI:** 10.3390/s110908813

**Published:** 2011-09-13

**Authors:** Mª Soledad García, Joaquín A. Ortuño, María Cuartero, Mustafa Salem Abuherba

**Affiliations:** Department of Analytical Chemistry, Faculty of Chemistry, University of Murcia, E-30100 Murcia, Spain; E-Mails: jortuno@um.es (J.A.O.); mcb2@um.es (M.C.); abumusalem@yahoo.es (M.S.A.)

**Keywords:** ion-selective electrode, ziprasidone, mixed solvents, pharmaceuticals, urine, serum

## Abstract

The construction and characterization of a new ion-selective electrode for the determination of the antipsychotic ziprasidone in mixed solvents is presented. The electrode contains a plasticized polymeric membrane based on a ziprasidone-tetraphenylborate ion-exchanger. The influence of membrane composition on the electrode response towards ziprasidone in hydroalcoholic solutions was studied. The electrode displayed a stable response in a 2:3 (v/v) methanol/water medium from a ziprasidone concentration of 3 × 10^−6^ M with a fast response time of less than 20 s. The electrode also showed good selectivity towards ziprasidone over common inorganic and organic compounds and several species with pharmacological activity. The electrode was successfully applied to the determination of ziprasidone in pharmaceuticals and human urine and serum.

## 1. Introduction

Many drugs are very hydrophobic and consequently poorly soluble in water, resulting in problems that may affect different fields of pharmaceutical science. The use of aqueous mixed solvents to increase the solubility of these drugs is one of the oldest, most powerful and widely used methods to address this issue [1].

The application of ion selective electrodes in mixed solvents involves a series of problems concerning membrane selection and the measuring technique to be used. However, potentiometric studies in these media have opened up new possibilities for analytical and thermodynamic fields based on the data obtained [2,3].

A thorough literature search revealed little information on the use of ion-selective electrodes (ISEs) based on plasticized polymeric membranes in mixed solvents. This is probably because the membrane components should exhibit low leaching into the sample solutions. Despite this, some authors have succeeded in using plasticized polymeric membrane ISEs for determining metal ions [4–24], alkaline and alkaline-earth ions [25], monohydrogen phosphate [26], chromate ions [27] and surfactants [28,29] in mixed solvents.

Ziprasidone (5-[2-[4-(1,2-benzisothiazol-3-yl)-1-piperazin-yl ethyl]-6-chloro-1,3-dihydro-2H-indol-2-one, ZPD, Figure 1), is a antipsychotic agent indicated for the treatment of schizophrenia, bipolar disorder and acute mania because of its affinity for serotonin (5HT2A) and dopamine (D2) receptors [30]. The therapeutic importance of this drug requires the development of sensitive and rapid analytical methods for its determination in pharmaceutical and clinical analysis. A review of the literature showed that several analytical methods have been described in this respect, including spectrophotometric [31–33], thin layer densitometry [34], capillary electrophoresis [35], voltammetric [36] and chromatographic [37–41] methods. However, to our knowledge, there is no ISE to determine ZPD. A possible reason for this could be the low solubility of ZPD in water, which makes it necessary to use non-aqueous or mixed solvents.

Since potentiometry with ISEs offers many advantages, among them simple design and operation, low cost, fast response and the possibility of automation, the aim of the present work was to develop a potentiometric selective electrode for ZPD determination which could be used in mixed solvents for application in pharmaceutical and clinical analysis.

## 2. Experimental Section

### 2.1. Apparatus

Potentials were measured with an Orion 960 Autochemistry System (Thermo-Orion, Cambridge, MA, USA), the recorder output of which was connected to a personal computer via a DGH Corporation 1121 module analogue-to-digital converter (Manchester, UK). An Orion 90–02 double junction silver-silver chloride reference electrode containing a 10% (v/v) solution of KNO_3_ in the outer compartment and a Fluka (Munich, Germany) ISE electrode body was used.

### 2.2. Reagents and Solutions

High molecular weight poly(vinyl chloride) (PVC), 2-nitrophenyl octyl ether (NPOE), bis(2-ethylhexyl) sebacate (DOS), and tetrahydrofuran (THF) were Selectophore products from Fluka (Munich, Germany). Sodium tetraphenylborate (NaTPB) was purchased from Sigma (Munich, Germany). Ziprasidone hydrochloride powder was kindly provided by Pfizer Laboratories (New York, USA). All other reagents used were of analytical reagent grade and Milli-Q water was used throughout. Ac^−^/HAc buffer of pH 4.6 and 2 × 10^−1^ M total concentration was used.

A 4 × 10^−3^ M ziprasidone hydrochloride standard solution was prepared by dissolving 0.0467 g of ziprasidone hydrochloride in 25 mL of methanol. Working solutions (8 × 10^−4^ M to 1.6 × 10^−6^ M) were prepared by diluting appropriate volumes of the standard solution with 15 mL of Ac^−^/HAc buffer of pH 4.6 and methanol to a final volume of 25 mL in a calibrated flask.

For the selectivity study, 4 × 10^−3^ M solutions of amoxicillin, carbamazepine, cimetidine, clomipramine hydrochloride, diclofenac sodium, fluoxetine hydrochloride, furosemide, haloperidol, lansoprazol, lorazepan, ranitidine, ofloxacine and tenoxicam were prepared by dissolving suitable amounts of the corresponding drug in methanol.

Dosage forms of ziprasidone for testing: Zeldox capsules (Pfizer Lb., Madrid, Spain) containing 20 mg of ziprasidone hydrochloride with lactose monohydrate, starch, magnesium stereate, gelatine, dioxidum of titanium (E171) and indigotine (E132) up to total capsule weight; Zeldox powder for injectable solution containing 20 mg of ziprasidone mesylate and sodium sulfobutylether-β-cyclo-dextrin up to total vial powder weight.

### 2.3. Ion-Exchanger Preparation

The ion-exchanger (ZPD-TPB) was prepared by mixing 10 mL of 4 × 10^−3^ M ZPD with 10 mL of a 4 × 10^−3^ M NaTPB aqueous solution. The mixture was filtered through a porosity-4 sintered glass crucible. The residue was washed with 1:1 (v/v) methanol/water solution until no chloride ion was detected in the washing solution and was then dried at room temperature.

### 2.4. Construction and Conditioning of the Electrode

The selected membrane was prepared by dissolving 3.0 mg of ZPD-TPB, 100 mg of PVC and 200 mg of NPOE in 3 mL of THF. This solution was poured into a Fluka glass ring (inner diameter 28 mm, height 30 mm) on a Fluka glass plate. The solution was allowed to evaporate overnight. A 7-mm diameter piece was cut out with a Fluka punch for ion-selective membranes and incorporated into a Fluka electrode body ISE containing 1 × 10^−2^ M KCl and 8 × 10^−5^ M ZPD saturated with AgCl as the internal filling solution. The electrode was conditioned by soaking with constant stirring in 8 × 10^−5^ M ZPD working solution until the electrode gave a constant potential. When not in use, the electrode was kept immersed in the same solution.

### 2.5. Calibration of the Electrode

The ZPD-selective electrode and reference electrode were immersed in any of the working ZPD solutions and the potential was measured, under constant stirring, until it gave a constant value. These potentials were then plotted *vs.* logarithmic values of ZPD concentration and the calibration parameters were calculated by fitting the calibration data to Equation (1):

(1) $$E=E^{0}+\mathrm{Slog}(\mathrm{LOD}+C_{\mathrm{ZPD}})$$

where E is the potential of the cell, E^0^ is the standard potential, S and LOD are the slope and the limit of detection of the electrode, respectively.

For the dynamic response studies a dynamic calibration of the electrode was made by adding, while stirring, adequate small volumes by micropipette of the ZPD standard solution to 25 mL of a 2:3 (v/v) methanol/pH buffer solution to cover the concentration range from 1.6 × 10^−6^ to 1.6 × 10^−4^ M.

### 2.6. Procedure for the Determination of Ziprasidone in Pharmaceuticals

The ZPD content in capsules was determined by analyzing four capsules separately. The powder content of each one was shaken with 15 mL of methanol and then placed in an ultrasonic bath for 15 min and finally diluted with methanol in a 25 mL calibrated flask. A portion of this solution was centrifuged at 3,000 rpm for 5 min and the supernatant was filtered through a filter paper. Different volumes (500–1,000 μL) of the filtrate were taken, to which 15 mL of buffer were added and finally diluted to 25 mL with methanol in a calibrated flask. A similar procedure was followed in the case of ZPD powder for injectable solutions. The ZPD concentration in the sample was determined by comparing the potential value measured with the calibration graph.

Recovery studies were performed by adding different volumes (125–400 μL) of the ZPD standard solution to 1.0 mL of the pharmaceutical sample solution. Then, 15 mL of pH buffer were added and the solution was diluted to 25 mL with methanol in a calibrated flask. The samples were analyzed in triplicate using the procedure described above.

### 2.7. Procedure for the Determination of Ziprasidone in Human Urine and Serum

For the determination of ZPD in human urine samples the sample pH was adjusted to 4.6 with HCl and NaOH solutions. Serum samples were first deproteinized by adding a volume of 8% trichloroacetic acid solution to the same volume of serum. Then, the solutions were shaken in a vortex mixer for 30 s, allowed to settle for 20 min and centrifuged for 10 min at 1,200 rpm. The clear supernatant layer was filtered through a 0.45 μm Millipore filter and the pH was adjusted to 4.5 by adding 2 M NaOH. In the absence of urine and serum samples containing ZPD, known amounts of ZPD were added. The potential was measured and compared with calibration graphs made in pooled urines and pooled serum, respectively.

## 3. Results and Discussion

### 3.1. Influence of Membrane Composition

Membranes of different compositions (Table 1), prepared as described in the Experimental section, were tested to optimize the membrane composition of the ZPD-electrode. Two plasticizers with different dielectric constants were used as membrane solvent, NPOE (ɛ = 23.9) and DOS (ɛ = 2.4), membranes A and B, respectively. The corresponding calibration graphs for ZPD obtained in a 2:3 (v/v) methanol/pH buffer medium are shown in Figure 1. As can be seen, the membrane plasticized with NPOE showed higher potential span and lower detection limit, an effect that agrees with reports in the literature for other drug ISEs [42,43]. The difference observed between both membranes can be explained by the fact that the detection limit of ISEs based on dissolved ion-exchangers may be governed by the analyte ion activity present in the sample solution as a result of the distribution equilibrium of the ion-exchanger between the membrane and the solution [44]. The solubility of the ion-exchanger in the membrane generally increases as the polarity and dielectric constant of the membrane increases [45], which implies a lower concentration in the sample solution and therefore a lower detection limit. Taking these results into account NPOE was selected as plasticizer for further studies.

Two different ZPD-TPB ion exchanger concentrations in the membrane were tested (membranes A and C, respectively). As can be seen in Figure 1, the membrane containing the higher ion-exchanger concentration displayed a poorer potential response. Taking all this into account, membrane A was selected for further studies.

### 3.2. Influence of the pH

**Figure 2.**
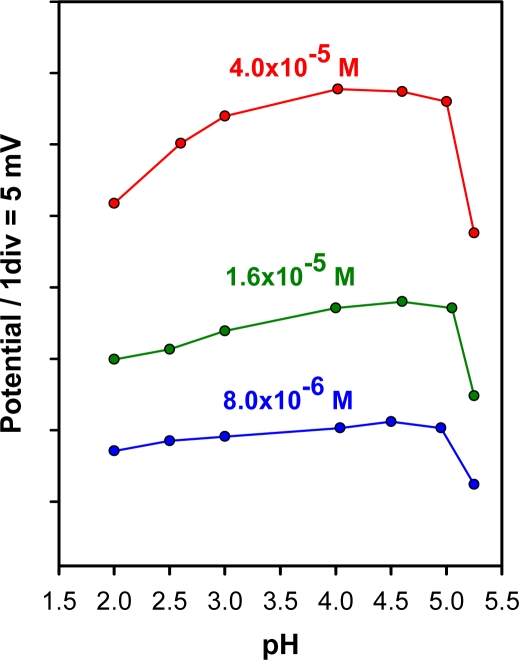
Influence of the pH on the electrode potential for different ziprasidone concentrations.

The effect of pH on the electrode potential at various ZPD concentrations in the range 8.0 × 10^−6^–4.0 × 10^−5^ M, was studied. The pH was varied by adding HCl or NaOH and the results are shown in Figure 2. As can be seen, the electrode potential was somewhat dependent on pH and therefore the use of a pH buffer was thought convenient. A pH of 4.6 adjusted with 2 × 10^−1^ M sodium Ac^−^/AcH buffer solution was used for further studies.

### 3.3. Influence of the Mixed Solvent Composition

**Figure 3.**
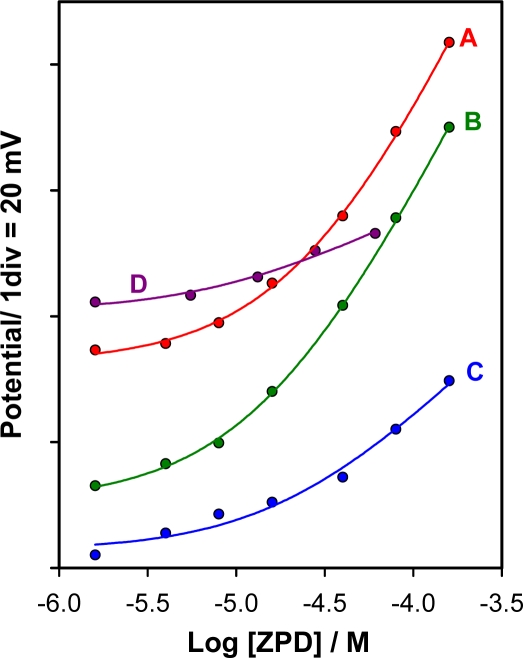
Calibration graphs in different media. (**A**) 1:4 (v/v) methanol/water; (**B**) 2:3 (v/v) methanol/water; (**C**) 3:2 (v/v) methanol/water; and (**D**) 2:3 (v/v) ethanol/water.

Since ZPD is soluble in methanol and ethanol, the performance of the electrode was evaluated in hydroalcoholic media, *i.e.*, methanol-water and ethanol-water mixtures of different ratios. All of them contained buffer solution of pH 4.6. Figure 3 shows the ZPD calibration graphs A-D determined in methanol/water 1:4, 2:3 and 3:2 (v/v), and ethanol/water 2:3 (v/v), respectively. By comparing the curves corresponding to different methanol contents, it is obvious that the best response was displayed when a methanol/water 2:3 (v/v) medium was used (curve B). When ethanol was used instead of methanol, the potential response was very poor. Accordingly to all this, a methanol/water 2:3 (v/v) medium was used for further studies.

### 3.4. Validation of the Analytical Method

The validation of the analytical method for the determination of ziprasidone based on the present ZPD-selective electrode was made following recommendations of the literature [46,47].

#### 3.4.1. Response Characteristics

**Figure 4.**
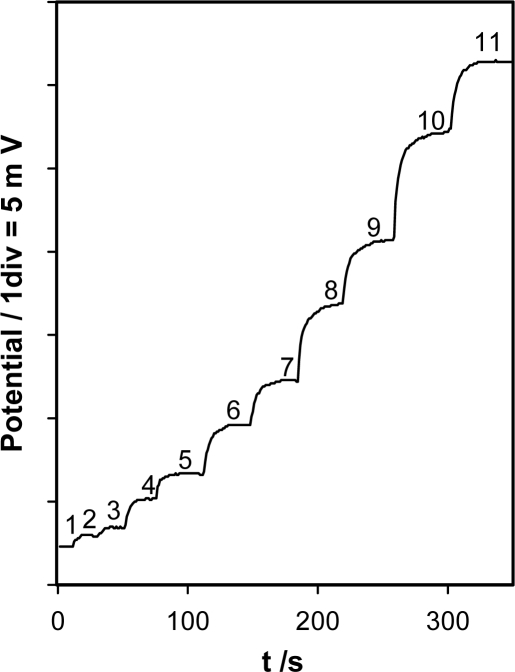
Dynamic response of the electrode for ziprasidone concentrations of (1) 0 M; (2) 1.6 × 10^−6^; (3) 3.2 × 10^−6^ M; (4) 7.2 × 10^−6^ M; (5) 1.1 × 10^−5^ M; (6) 1.9 × 10^−5^ M; (7) 2.7 × 10^−5^ M; (8) 4.3 × 10^−5^ M; (9) 5.8 × 10^−5^ M; (10) 9.7 × 10^−5^ M; and (11) 1.6 × 10^−4^ M.

The slope and the limit of detection (LOD) of the selected electrode were determined by non-linear curve fitting of the corresponding calibration data to Equation (1). The values obtained for the range, linearity, slope, limit of detection and accuracy together with the response time of the electrode obtained from a dynamic calibration (Figure 4), are shown in Table 2. As can be seen, there was a Nernstian response over almost two concentration decades, with a low detection limit and a rapid response, even at low ZPD concentrations, was obtained.

#### 3.4.2. Repeatability and Reproducibility

The repeatability and reproducibility of the calibration parameters were studied by making successive calibrations with the same membrane on the same day (n = 6), with the same membrane on different days (n = 6) and with different membranes (n = 3). The results obtained for the LOD were 1.8 × 10^−5^ ± 2.1 × 10^−6^ M, 2.2 × 10^−5^ ± 7.5 × 10^−6^ M and 2.8 × 10^−5^ ± 1.1 × 10^−5^ M, respectively, and for the slopes, 59.3 ± 1.8, 58.4 ± 2.7 and 59.2 ± 2.8 mV per decade of concentration, respectively. In all these aspects, a reasonably good reproducibility was obtained.

#### 3.4.3. Electrode Lifetime

The electrode lifetime was obtained by periodically performing calibration graphs for ZPD and calculating the response parameters. The ZPD selective electrode worked for at least 40 days, during which time no appreciable change in the calibration characteristics or response time was observed. After this time, the slope of the electrode started to decrease.

#### 3.4.4. Selectivity

The response of the ZPD-selective electrode was studied toward K^+^, NH_4_^+^, Ca^2+^, Mg^2+^, glucose, lactose, saccharose, urea, uric acid, hipuric acid, amoxicillin, cimetidine, ofloxacin, diclofenac, tenoxicam, lansoprazole, loracepam, carbamazepine, fluoxetine, biperidene, haloperidol, and ranitidine. This list includes species frequently present in pharmaceuticals and biological fluids and drugs that could be administered at the same time as ZPD. Solutions of these compounds were prepared in methanol and the corresponding calibration graphs were carried out in the selected medium. The electrode did not respond to K^+^, NH_4_^+^, Ca^2+^, Mg^2+^, glucose, lactose, saccharose, urea, uric acid, hipuric acid, amoxiciline, cimetidine, ofloxacin, diclofenac, tenoxicam, lansoprazole, loracepam, carbamazepine and ranitidine at concentrations below 3 × 10^−4^ M. The electrode displayed a near Nernstian response to fluoxetine, haloperidol and biperidene and so the selectivity coefficients of these three species were determined by applying the separate solution method [48]. The values obtained were 1.5, 0.5 and 2.4, respectively.

### 3.5. Analytical Applications

In order to demonstrate the applicability of the proposed method to the determination of ZPD, the method was applied to its determination in pharmaceuticals and human urine and serum. Two different pharmaceutical dosage forms of ZPD were analysed following the method described in the Experimental (Table 3). In the absence of a reference method for the analytical determination of ZPD in the literature, recovery studies were carried out (Table 3), providing quantitative recoveries between 99.3 and 101.7% in all the cases.

For the determination of ZPD in human urine, previous calibrations using different urines were made. No significant differences between the corresponding calibration parameters were found. Therefore, a calibration made using pooled urine samples was used for the determination of ZPD in different samples. A similar procedure was used for the determination of ZPD in serum. The results obtained are shown in Table 4. Good recoveries were obtained for the determination of ZPD in both types of biological sample.

The systemic bioavailability of ZPD administered intramuscularly is 100% and 60% if it is administered orally. After a single dose intramuscular administration, the peak serum concentration typically occurs at about 60 minutes after the dose is administered, or earlier. In the case of an oral administration, the peak serum concentration is reached from 2 to 6 hours after the administration of therapeutic doses and approximately 44% of the total serum ZPD remains unchanged. The normal therapeutic dose of ZPD recommended for adults is between 40 and 160 mg/day and from 1 to 4% is excreted unchanged in the urine after its administration. Taking into account these data and the results obtained, it can be concluded that the determination of ZPD in human urine and serum is feasible using the present electrode being this method very useful for routine clinical analysis.

### 3.6. Advantages of the Proposed Method

The potentiometric method proposed shows some advantages over the other electroanalytical method reported in the literature [36] which makes use of voltammetric techniques. Thus, while the voltammetric method requires electrode polishing before each experiment, no electrode pre-treatment is needed in the present potentiometric method. Variations of the pH of the medium also have less influence in the potentiometric method, which is not affected by the presence of some other drugs and compounds frequently present in pharmaceuticals and biological fluids. Finally, while both methods are useful for pharmaceuticals and serum analysis, the potentiometric method was also applied to urine analysis.

## 4. Conclusions

The presented ISE provides a rapid, sensitive, reproducible and precise method for the potentiometric determination of ziprasidone in mixed solvents. The new electrode could be very useful in pharmaceutical analysis in the absence of a existing reference method for ziprasidone determination. The electrode can also be used to determine ziprasidone in human urine and serum.

## Figures and Tables

**Figure 1.**
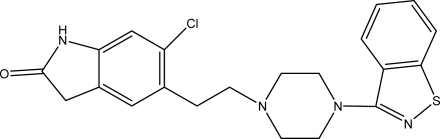
Ziprasidone.

**Figure 1.**
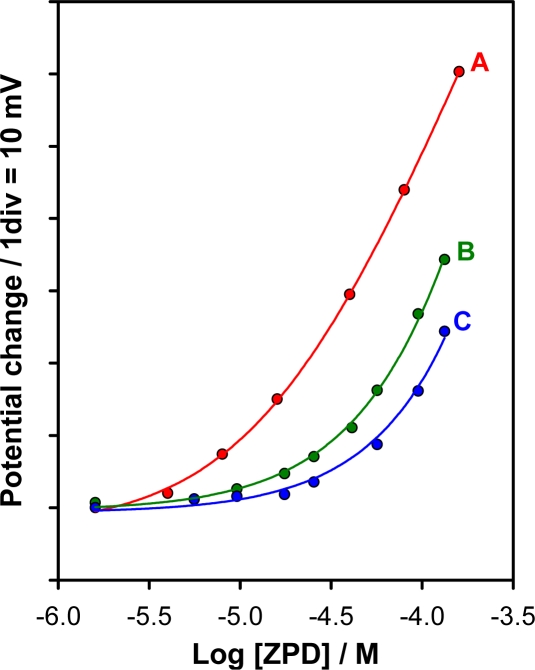
Calibration graphs of ziprasidone obtained with membranes A, B and C.

**Table 1.** Composition of the membranes assayed.

	**Percentage (w/w) of components in membranes**			

**Membrane**	PVC	NPOE	DOS	ZPD-TPB
A	33.0	66.0		1.0
B	33.0		66.0	1.0
C	32.3	64.7		3.0

**Table 2.** Response characteristics of the ziprasidone selective electrode.

**Parameter**	**Value**
Range (M)	1.6 × 10^−6^–1.6 × 10^−4^
Linearity (M)	2 × 10^−5^–8 × 10^−4^
Slope (mV decade^−1^) ± RSD ^a^	59.3 ± 1.8
Limit of detection (M) ± RSD ^a^	1.8 × 10^−5^ ± 2.1 × 10^−6^
Accuracy ^c^ (%)	99.38 ± 0.37
Response time (s) ^b^	t_95%_ ≤ 15
Working pH range ^b^	3.5–5.5
Operational lifetime (days)	≥ 40

a mean of six calibrations.

b ZPD concentration range of 1.6 × 10^−6^–1.6 × 10^−4^ M.

c Recovery ± RSD (n = 10).

**Table 3.** Determination of ziprasidone in pharmaceuticals.

**Sample**	**Labelled^a^**	**Amount found^a^**	**% Bias**	**Recovery studies**			
				**Added^a^**	**Found^a^**	**Recovery^c^ (%)**	**% Bias**
Zeldox capsules	20.00	20.02 ± 0.54 ^b^	0.10	5.86	25.82 ^b^	99.3 ± 0.5	−0.68
				11.73	31.93 ^b^	101.7 ± 4.1	1.70
				18.70	38.68 ^b^	99.9 ± 3.5	−0.11

Zeldox injectables	20.00	20.39 ± 1.09 ^b^	1.90	5.86	25.87 ^b^	100.2 ± 0.7	0.17
				11.73	31.80 ^b^	100.6 ± 0.2	0.59
				18.70	38.66 ^b^	99.8 ± 0.1	−0.21

a ZPD mg/capsule or injectable.

b Average of three experiments.

c Mean ± RSD (n = 4).

**Table 4.** Determination of ziprasidone in human urine and serum.

**Sample**	**Amount added**	**Found^c^**	**Recovery^c^ ± RSD (%)**	**% Bias**
Urine 1	2.80^a^	2.70	96.4 ± 1.2	−3.57
Urine 2	4.70^a^	4.73	100.6 ± 0.5	0.64
Urine 3	8.30^a^	8.08	97.4 ± 2.0	−2.65
Urine 4	12.0^a^	12.3	102.5 ± 1.4	2.50
Serum 1	0.90^b^	0.93	103.3 ± 1.1	3.33
Serum 2	2.80^b^	2.79	99.6 ± 0.3	−0.36
Serum 3	6.50^b^	6.49	99.8 ± 0.2	−0.15
Serum 4	15.6^b^	15.88	101.8 ± 0.9	1.79

a ZPD mg/L urine.

b ZPD μg/mL serum.

c Average of five experiments.
